# Deducing the stage of origin of Wilms' tumours from a developmental series of *Wt1*-mutant mice

**DOI:** 10.1242/dmm.018523

**Published:** 2015-08-01

**Authors:** Rachel L. Berry, Derya D. Ozdemir, Bruce Aronow, Nils O. Lindström, Tatiana Dudnakova, Anna Thornburn, Paul Perry, Richard Baldock, Chris Armit, Anagha Joshi, Cécile Jeanpierre, Jingdong Shan, Seppo Vainio, James Baily, David Brownstein, Jamie Davies, Nicholas D. Hastie, Peter Hohenstein

**Affiliations:** 1 MRC Human Genetics Unit, MRC Institute for Genetics and Molecular Medicine, University of Edinburgh, Western General Hospital, Crewe Road, Edinburgh, EH4 2XU, UK; 2 The Roslin Institute, University of Edinburgh, Easter Bush Campus, Midlothian, EH25 9RG, UK; 3 Department of Biomedical Informatics and Developmental Biology, Cincinnati Children's Hospital Medical Center, Cincinnati, OH 45229, USA; 4 INSERM, UMR 1163, Laboratory of Inherited Kidney Diseases, Paris 75015, France; 5 Paris Descartes – Sorbonne Paris Cité University, Imagine Institute, Paris 75015, France; 6 Biocenter Oulu, InfoTech Oulu, Faculty of Biochemistry and Molecular Medicine, Aapistie 5A, University of Oulu, PO Box 5000, Oulu 90014, Finland; 7 Queen's Medical Research Institute, University of Edinburgh, 47 Little France Crescent, Edinburgh, EH16 4TJ, UK; 8 Centre for Integrative Physiology, University of Edinburgh, Hugh Robson Building, 15 George Square, Edinburgh, EH8 9XD, UK

**Keywords:** WT1, Wilms’ tumour, Kidney development, Mouse model

## Abstract

Wilms' tumours, paediatric kidney cancers, are the archetypal example of tumours caused through the disruption of normal development. The genetically best-defined subgroup of Wilms' tumours is the group caused by biallelic loss of the *WT1* tumour suppressor gene. Here, we describe a developmental series of mouse models with conditional loss of *Wt1* in different stages of nephron development before and after the mesenchymal-to-epithelial transition (MET). We demonstrate that Wt1 is essential for normal development at all kidney developmental stages under study. Comparison of genome-wide expression data from the mutant mouse models with human tumour material of mutant or wild-type *WT1* datasets identified the stage of origin of human *WT1*-mutant tumours, and emphasizes fundamental differences between the two human tumour groups due to different developmental stages of origin.

## Abstract (precis)

**Summary:** The comparison of different nephron-specific *Wt1*-knockout mouse models identifies the stage of origin of human *WT1*-mutant Wilms' tumours.

## INTRODUCTION

Wilms' tumours (Wilms, 1899) are paediatric kidney cancers that affect 1:10,000 children, usually before the age of five (recently reviewed in Hohenstein et al., 2015). They are thought to be caused by a block in the normal development of nephrons, as illustrated by the appearance of nephrogenic rests – structures resembling embryonic renal tissues and believed to be the first stage of Wilms' tumour development. Therefore, to truly understand the development of Wilms' tumour, a full understanding of normal kidney development and the role of genes linked to Wilms' tumorigenesis in this process is essential.

The developing metanephric kidney is a model system for many developmental processes, including reciprocal inductions between different cell types, mesenchymal-to-epithelial transitions (METs) and patterning (Saxen, 1987). The initiating event in metanephric kidney development is the invasion of the ureteric bud, an epithelial outgrowth of the Wolffian duct, into the metanephric mesenchyme, which is a derivative of the intermediate mesoderm. In response, signals are exchanged bidirectionally between these populations, resulting in the first branching of the ureteric bud and the formation of a condensate of mesenchymal cells, the cap mesenchyme, around the tips of the ureteric bud. Expression of *Six2* in the cap mesenchyme labels the nephron progenitor cells (Kobayashi et al., 2008). A canonical Wnt signal, probably emanating from Wnt9b expressed in the ureteric bud, determines which of the *Six2*-positive cells remain in the progenitor cell state and which will differentiate to form the nephron (Karner et al., 2011; Park et al., 2012). A Yap-Fat4-mediated signal originating from *Foxd1*-positive stromal cells further controls this decision (Das et al., 2013). The cells that are induced to differentiate undergo a MET under control of *Wnt4* (Kispert et al., 1998; Stark et al., 1994) to form the epithelialized renal vesicle, probably mediated by the non-canonical Wnt-Ca^2+^-NFAT pathway (Burn et al., 2011; Tanigawa et al., 2011). Disruption of this MET is believed to be a central event in the development of Wilms' tumours (Hohenstein et al., 2015). The renal vesicle becomes patterned along the proximal-distal axis, connects to the ureteric bud at its distal end and, through the comma- and S-shaped body stages, develops into the mature nephron, which consists of distal tubule, loop of Henle, proximal tubule and the glomerulus, containing the filtering podocytes. This nephron induction, differentiation and maturation process is repeated every time the ureteric bud branches and new tips are formed.

Wilms' tumours were one of the cancers that Alfred Knudson used to develop the two-hit model and concept of tumour suppressor genes (Knudson and Strong, 1972). This led to the identification of the *WT1* tumour suppressor gene, loss of which is the rate-limiting step in 15% of Wilms' tumours (Hohenstein et al., 2015). This subset of cases is characterized by ectopic muscle development (Miyagawa et al., 1998; Schumacher et al., 2003) and selection for activating mutations in *CTNNB1*, the gene encoding β-catenin (Koesters et al., 1999; Maiti et al., 2000). Accordingly, the *WT1* wild-type and mutant subsets of tumours can clearly be recognized using genome-wide expression analysis (Corbin et al., 2009; Gadd et al., 2012; Li et al., 2004). The genetics of *WT1*-wild-type tumours are less clear. Activation of the *IGF2* pathway through loss of heterozygosity (LOH) or loss of imprinting (LOI) has been identified in many of these cases, but it is unclear whether this is the initiating event in these cases (Hohenstein et al., 2015). *WTX* was identified as a Wilms' tumour gene on the X chromosome (Rivera et al., 2007), but the details of involvement of *WTX* loss in the origins of Wilms' tumours have been disputed (Perotti et al., 2008; Rivera et al., 2007; Wegert et al., 2009). In all, *WT1* remains the best genetic and molecular entry point to study the origins of and mechanisms leading to Wilms' tumours.

**TRANSLATIONAL IMPACT**

**Clinical issue**

Wilms’ tumours are a form of childhood kidney cancer that originate from problems during prenatal kidney development. In humans, different subgroups of tumours have been described that show different responses to therapy and have different clinical outcomes. The genetically best-defined tumour subtypes are associated with expression of either the wild-type or mutant (bearing loss-of-function mutations) Wilms’ tumour-1 (*WT1*) gene, a tumour suppressor gene. Identifying the developmental stage of origin of Wilms’ tumours will allow the design of new and improved therapies based on a better understanding of normal and impaired kidney development. Genome-wide expression analysis of human tumours has suggested that different tumour subsets originate during different developmental stages, but this has not been experimentally validated yet.

****Results****

In this study, the authors used a conditional *Wt1*-knockout mouse model to inactivate the gene during three different stages of kidney development, before and after the mesenchymal-to-epithelial transition (MET). They found that losing Wt1 at each of these stages results in the disruption of kidney development at specific stages and death of the pups immediately after birth. Then, the authors compared the genome-wide expression patterns of the mouse mutant kidneys to the expression patterns of human Wilms’ tumours bearing either mutant or wild-type *WT1*. Interestingly, the analyses showed that the expression data from the mouse mutants in which nephron development is blocked before the MET most closely resemble those of the human *WT1*-mutant tumours, whereas the data from the mouse mutants with a post-MET block more closely resemble the expression pattern found in human *WT1*-wild-type tumours.

****Implications and future directions****

This study suggests that Wt1 is an essential regulator of kidney development. In addition, the comparison of genome-wide expression data from the mouse mutants with human tumour datasets suggests that different human Wilms’ tumour subtypes have different developmental stages of origin. Caution should be taken in the interpretation of these data, because the developmental stages studied in the current experimental settings might naturally differ from the ones deduced from human tumour gene expression data. Nevertheless, these findings change our understanding of the biological causes of Wilms’ tumours and will help identify the biological pathways that could be targeted in different subgroups of tumours for therapeutic purposes.

*WT1* is expressed throughout nephron development (Armstrong et al., 1993; Pritchard-Jones et al., 1990). The earliest expression in the renal lineage is found in the intermediate mesoderm. Subsequently, expression is found in the metanephric mesenchyme and cap mesenchyme. After the MET, its expression becomes restricted to the proximal end of the developing nephron until, in the mature nephron, it is only found in the podocytes. The *WT1* gene encodes a collection of at least 36 multifunctional isoforms involved in translation, RNA metabolism and transcriptional control (Hohenstein and Hastie, 2006). In the intermediate mesenchyme it has a pro-survival function: these cells go into complete apoptosis in the conventional *Wt1*-knockout mouse (Kreidberg et al., 1993). In later stages, the gene has been linked to control of the MET (Davies et al., 2004; Essafi et al., 2011). Wt1 has essential functions in the development of podocytes, as shown in mouse models and human syndromes (Gao et al., 2004; Miller-Hodges and Hohenstein, 2012; Ozdemir and Hohenstein, 2014), as well as in the maintenance and function of mature podocytes, as we showed by knocking out *Wt1* in adult mice (Chau et al., 2011). However, the role of Wt1 in the developmental stages between cap mesenchyme and podocyte is less clear.

To start elucidating the events that lead to Wilms' tumour formation, we analyzed the role of Wt1 before and after the MET, one of the candidate stages of origin of the tumours. Our data show that the loss of *Wt1* at different stages during nephron development results in different phenotypes. Comparison of genome-wide expression data of these mutants to Wilms' tumour microarray data suggests the stages of origin of the *WT1*-mutant and *WT1*-wild-type tumour subsets, and further highlights their different developmental characteristics.

## RESULTS

### Loss of Wt1 with different nephron Cre drivers results in different renal phenotypes

**Fig. 1.**
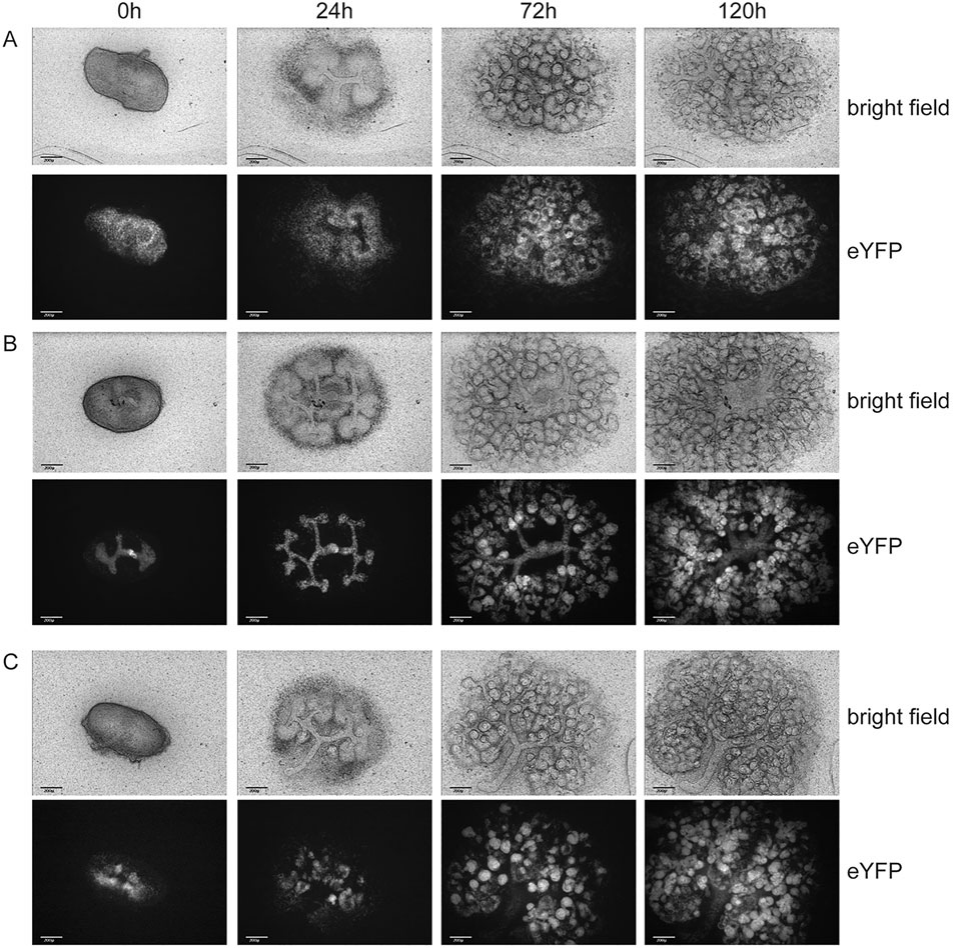
**Lineage tracing of three Cre drivers in cultured kidney rudiments starting from E11.5 kidneys for the indicated time intervals.** (A) *Nes*-Cre *Rosa26*^eYFP/eYFP^. (B) *Pax8*^+/Cre^ *Rosa26*^eYFP/eYFP^. (C) *Wnt4*^+/CreGFP^ *Rosa26*^eYFP/eYFP^. Scale bars: 200 µm.

To knock out *Wt1* in different cell types during renal development, we selected three Cre strains and characterized them in the embryonic kidney through lineage tracing using time-lapse imaging (Watanabe and Costantini, 2004) and an eYFP-based Cre reporter (Srinivas et al., 2001). *Nes*, the gene encoding the intermediate filament protein Nestin, is expressed at least as early as E12.5 in the embryonic kidney mesenchyme and is probably a direct transcriptional target for Wt1 (Wagner et al., 2006). Accordingly, the *Nes*-Cre allele that we used (Tronche et al., 1999) showed widespread activity in the mesenchyme at E11.5. During subsequent culturing, the lineage trace was restricted to the nephrogenic lineage and excluded from the ureteric bud (Fig. 1A and supplementary material Movie 1A). Initially, there was a parallel and evenly spaced movement of labelled cap mesenchymes, as if being ‘pushed out’ by the ureteric bud; later, during the cultures, there seemed to be a steady stream of labelled cells from the outside of the growing rudiment into the kidney. Use of a Cre allele expressed from the endogenous *Pax8* locus (Bouchard et al., 2004) resulted in scattered eYFP-positive cells in the E11.5 metanephric mesenchyme and a strong signal in the condensed mesenchyme and in the ureteric bud (Fig. 1B and supplementary material Movie 1B). During the subsequent culture, the lineage trace was found extensively in the newly formed condensates, the resulting nephrons and the ureteric bud. The time-lapse data illustrate how some labelled cells from the cap mesenchyme are left behind by the growing bud, increase in number and condense to form a nephron. Because *Wt1* is not expressed in the ureteric bud, the activity of *Pax8*^+/Cre^ there does not affect the analysis presented here. Finally, we used a Cre-GFP fusion construct knocked into the endogenous *Wnt4* locus (Shan et al., 2010). As described, this driver becomes active in the pre-tubular aggregates and subsequently traces the complete nephron (Fig. 1C and supplementary material Movie 1C). The GFP signal from the *Wnt4*^CreGFP^ allele is barely detectable on the time-lapse system (Shan et al., 2010 and data not shown); therefore, all signal comes from the eYFP reporter.

Before crossing our *Wt1* conditional allele (Martinez-Estrada et al., 2010) to the three Cre drivers, we confirmed that it is a conditional version of the conventional *Wt1* knockout (Kreidberg et al., 1993) by crossing it to a germline Cre deleter strain expressing a CAGGs-driven Cre transgene to generate heterozygous *Wt1*^+/−^ mice. Homozygosity for this recombined allele was found to be an exact phenocopy of the conventional knockout, including the kidney, gonad, epicardium and diaphragm phenotypes (supplementary material Fig. S1 and data not shown). Next, the *Wt1* conditional allele was crossed to each of the three Cre drivers we had characterized above. Cre-positive (all three drivers)/*Wt1* conditional heterozygous mice were viable and healthy (data not shown). With one exception (see below), the Cre-positive *Wt1* conditional homozygous genotypes were not compatible with postnatal life. Embryos developed up to birth and were macroscopically normal (although slightly smaller than control littermates in the case of *Nes*-Cre *Wt1*^co/co^ embryos), but died immediately after birth. Because there is overlap between the activities of the Cre drivers and *Wt1* outside the kidney, we used optical projection tomography (OPT) (Sharpe et al., 2002) to generate 3D reconstructions of E16.5 whole embryos. The complete OPT image sets (3D volumes) with a number of visualizations and movies are provided on the eMouseAtlas (Richardson et al., 2014) community pages at http://www.emouseatlas.org/emap/community/submission000001/hohenstein.html. The OPT images can be viewed in virtual section mode using the IIP3D image viewer (Husz et al., 2012).

**Fig. 2.**
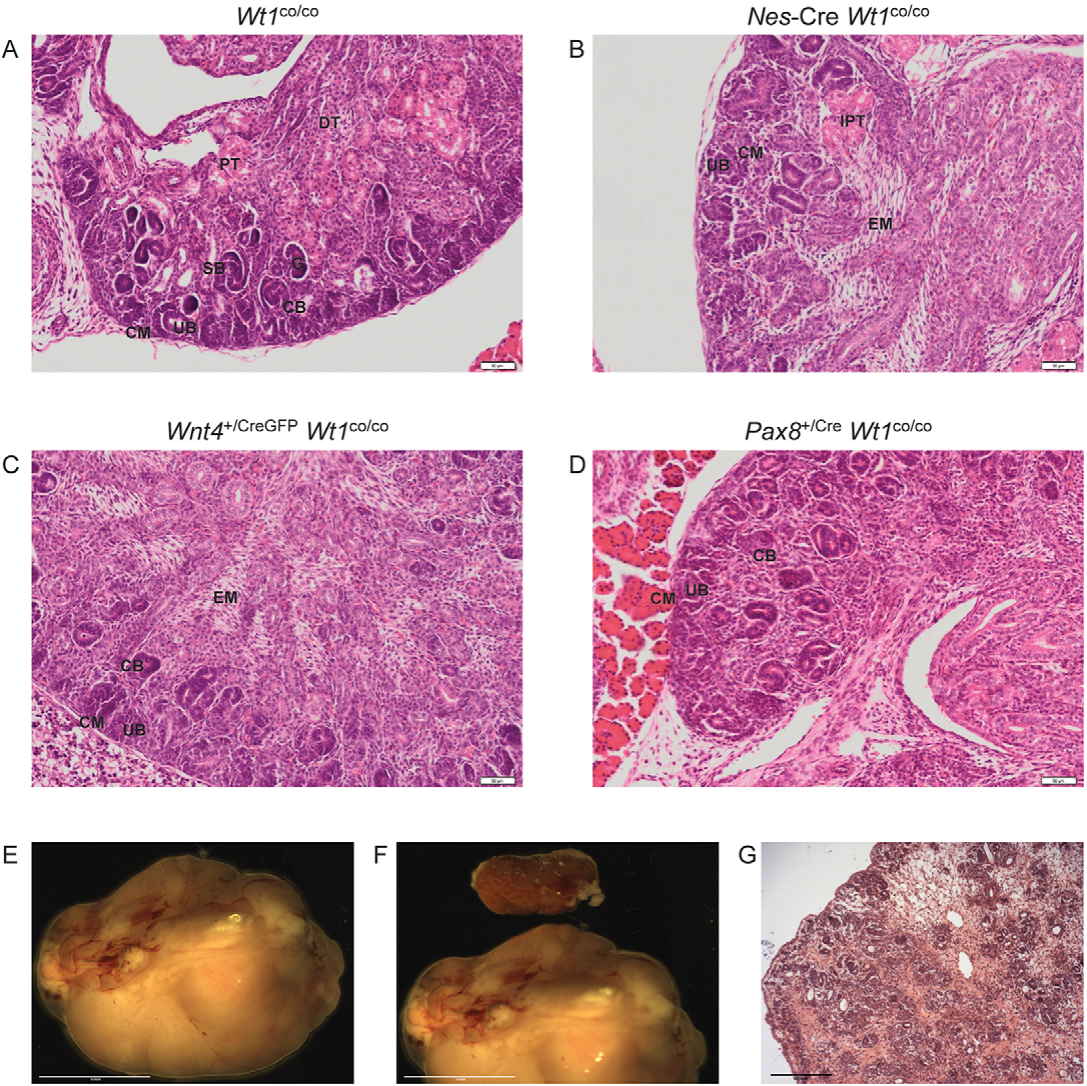
**Renal phenotypes in the three Cre *Wt1* conditional models.** (A-D) H&E-stained E18.5 embryonic kidneys. (A) *Wt1*^co/co^. (B) *Nes*-Cre *Wt1*^co/co^. (C) *Wnt4*^+/CreGFP^ *Wt1*^co/co^. (D) *Pax8*^+/Cre^ *Wt1*^co/co^. CM, cap mesenchyme; CB, comma-shaped body; SB, S-shaped body; UB, ureteric bud; PT, proximal tubule; DT, distal tubule; EM, expanded mesenchyme; IPT, immature proximal tubule. Scale bars: 50 µm. (E) Macroscopic view of Wilms’ tumour in single surviving *Nes*-Cre *Wt1*^co/co^ mouse. Scale bar: 10 mm. (F) Same tumour as in E with left kidney from the same mouse. Scale bar: 10 mm. (G) H&E staining of the tumour in E and F. Scale bar: 500 µm.

We analyzed the kidneys of E18.5 embryos in more detail (Fig. 2A-D). As described above, in *Nes*-Cre *Wt1*^co/co^ (homozygous *Wt1* conditional with *Nes*-Cre driver) kidneys, nephron development is disturbed at the MET stage, leading to an expansion of the mesenchyme. Condensation and epithelialization are severely affected, except for regions where *Wt1* is not lost owing to incomplete function of *Nes*-Cre (Essafi et al., 2011). In contrast, in *Wnt4*^+/CreGFP^ *Wt1*^co/co^ kidneys, MET is possible as there is condensation, epithelialization and early tubulogenesis (comma- and S-shaped body stages), albeit at reduced levels. Expanded mesenchyme can be found in these kidneys as well, although glomerulogenesis and tubular maturation are absent. *Pax8*^+/Cre^ *Wt1*^co/co^ kidneys showed normal levels of condensation, epithelialization and early tubulogenesis, but tubule maturation and glomerulogenesis were still absent. As was the case for the *Nes*-Cre *Wt1*^co/co^ kidneys (Essafi et al., 2011), no differences in proliferation or apoptosis were found in the other two models (data not shown); the ‘escaping nephrons' that developed completely normally but were still Wt1-positive in the *Nes*-Cre-driven mutant were not found in the other mutants either (data not shown).

We serendipitously identified one surviving *Nes*-Cre *Wt1*^co/co^ mouse. This mouse developed a lump on its back at 25 weeks of age. Macroscopic analysis identified a large tissue mass in place of the right kidney (Fig. 2E,F). Microscopic analysis identified this as a Wilms' tumour (Fig. 2G). The left kidney was relatively normal and contained many more escaping nephrons than usual (data not shown). We assume this left kidney provided sufficient renal function for this animal to survive, thereby giving the right kidney time to develop this tumour.

### Histological phenotyping of *Wt1* mutants through genome-wide expression analysis

**Fig. 3.**
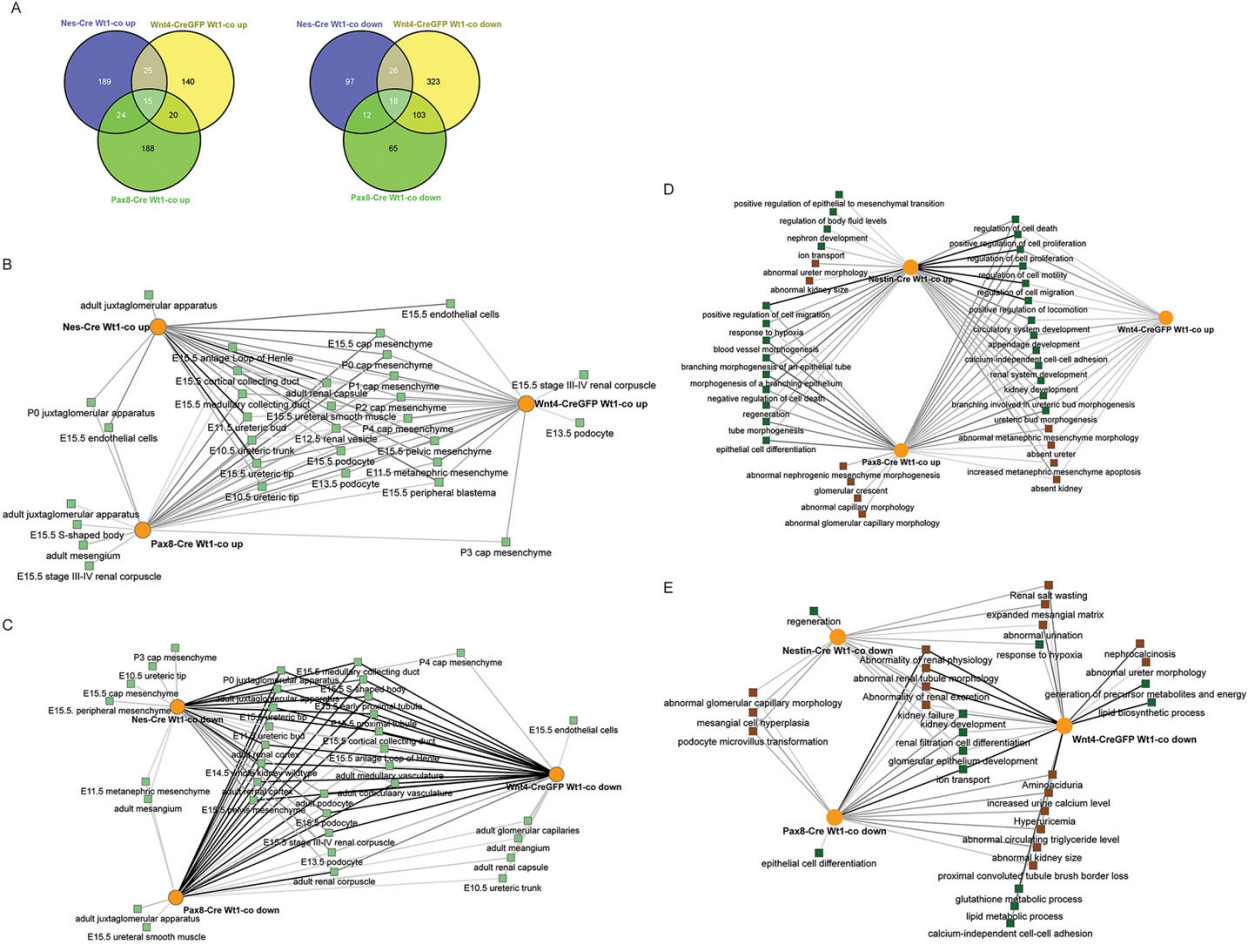
**Genome-wide expression analysis of E18.5 *Nes*-Cre *Wt1*^co/co^, *Wnt4*^+/CreGFP^*Wt1*^co/co^ and *Pax8*^+/Cre^*Wt1*^co/co^ kidneys.** (A) Comparison at the gene level for genes with increased (left panel) and decreased (right panel) expression. *Nes*-Cre *Wt1*^co/co^ differential genes are shown in blue, *Wnt4*^+/CreGFP^ *Wt1*^co/co^ differential genes in yellow and *Pax8*^+/Cre^ *Wt1*^co/co^ differential genes in green. (B) Enrichment for gene sets from cell type/developmental stage-specific GUDMAP datasets of increased genes in the mutant samples. (C) Enrichment for gene sets from cell type/developmental stage-specific GUDMAP datasets of decreased genes in the mutant samples. (D) Enrichment for biological processes (green nodes) and human/mouse phenotypes (brown nodes) coupled to genes increased in the mutant genotypes. (E) Enrichment for biological processes (green nodes) and human/mouse phenotypes (brown nodes) coupled to genes decreased in the mutant genotypes.

It has previously been shown that many genes are expressed in more than one developmental stage in the kidney (Brunskill et al., 2008). The identification of true anchor genes, which are uniquely expressed in just one specific cell type or developmental stage, can be challenging, especially for the stages immediately before and after the MET, on which we focused our analysis (Thiagarajan et al., 2011). This limits the usefulness of analyzing individual genes to describe developmental phenotypes like the ones found here. Instead, we hypothesized that global expression patterns could provide a means to describe complex phenotypes. To test this we generated microarray data from E18.5 total kidney RNA of the three mutant genotypes. We first compared each Cre^+^ *Wt1*^co/co^ dataset to the corresponding Cre^+^-only data to identify differentially expressed genes for each mutant and correct for changes due to expression of the Cre or, in the case of *Wnt4-* and *Pax8*-driven Cre alleles, haploinsufficiency of the driver gene (supplementary material Fig. S2 and Table S1). Comparison of transcripts with increased or decreased expression for each of the mutants showed 84 increased and 159 decreased genes were shared between two or three mutants, with larger numbers of genes being uniquely differentially expressed in individual mutants (Fig. 3A). To identify candidate direct targets for Wt1 in the differentially expressed genes, we compared the gene lists to *Wt1* ChIP-seq data from a recently published study on E18.5 mouse kidneys (Motamedi et al., 2014) and identified multiple genes in each differential gene set that showed *in vivo* binding of Wt1 to their genomic loci (supplementary material Table S2). Although experimental confirmation of these potential target genes falls outside the scope of this work (see Discussion), potentially important kidney developmental and disease genes can be identified this way. For instance, expression of receptor-type tyrosine-protein phosphatase O (*Ptpro*) is decreased in all three mutants and has direct binding of Wt1 to its genomic locus, making it a strong candidate for direct control by Wt1. This gene has previously been shown to be mutated in childhood-onset nephrotic syndrome (Ozaltin et al., 2011) and is a candidate gene for the *HIVAN4* nephropathy-susceptibility locus (Prakash et al., 2011).

We reasoned that decreased genes in these differential datasets would represent genes from kidney developmental stages that are not reached in the mutants due to the failure of maturation, whereas increased genes could be either enrichment of the genes expressed at the stage of the differentiation block or ectopically expressed genes. We compared our differential gene sets to the kidney-compartment-specific genome-wide expression patterns as they were generated (via microarray and RNA-seq analysis of micro-dissected and FACS-sorted cell populations) and made available by the GUDMAP project (Harding et al., 2011). These data provide expression signatures of each compartment in the developing kidney, even if the genes that make up this signature are not unique for this compartment. Note that, in this approach, we use differentially expressed genes solely as a signature for the stage at which development is blocked, and that this is independent from whether or not the differential genes are direct targets for control by Wt1. Gene-set enrichment analysis using the ToppCluster tool (Kaimal et al., 2010), which analyzes enrichment for each of these compartment-specific signatures (as well as to other biological datasets such as GO terms and phenotypic data sources), was used to further analyze the stage of developmental block in the different mutants. We hypothesized that enrichment for certain compartment-specific signatures would be indicative of a developmental block at that stage or close to that stage. The increased genes in the three mutants significantly overlapped with many genes from early developmental stages from the GUDMAP data (Fig. 3B). These include different cap mesenchyme datasets (including E15.5 and early postnatal), E11.5 metanephric mesenchyme and E15.5 pelvic mesenchyme. This demonstrates that the three mutants expressed sets of genes that are normally (in wild-type kidneys) found in these cell types and developmental stages. More informative were the enrichment patterns that were found for one or two of the mutants, because they illustrated the order in which the phenotypes arise. For instance, enrichment for genes normally expressed in the S-shaped body was exclusively found in the *Pax8*^+/Cre^ *Wt1*^co/co^ mutant kidneys. This is indeed the only one of the three mutants that can reach this developmental stage. Enrichment for genes normally found in the stage III and IV renal corpuscle (as defined on http://gudmap.org/Organ_Summaries/component_summary.php?componentID=10) can be recognized in the *Wnt4*^+/CreGFP^- and *Pax8*^+/Cre^-driven mutants. The *Nes*-Cre and *Pax8*^+/Cre^ *Wt1*^co/co^ mutants were enriched for genes normally expressed in the *Ren1*-positive cells from the adult juxtaglomerular apparatus, suggesting that these cells maintain a relatively primitive character. The enrichment for genes normally found in the adult mesangium in the *Pax8*^+/Cre^-driven mutants suggests an origin around the stage at which these mutants arrest for this cell type. For the decreased genes, enrichment is strongest for all late developmental and adult cell types in all three mutants (Fig. 3C), consistent with the fact that these stages are not reached in any of the models.

### Functional phenotyping of *Wt1* mutants through genome-wide expression analysis

Gene-set enrichment analysis of the differentially expressed genes performed using ToppGene (Chen et al., 2009) confirmed diverse effects of the three mutants (supplementary material Table S3). For instance, for the increased genes, the most significant ‘Molecular Function’ GO term was ‘receptor binding’ for the *Nes*-Cre *Wt1* conditionals (44 genes, *P*=9.24E–09), ‘platelet-derived growth factor binding’ (3 genes, *P*=2.20E–04) for the *Wnt4*-CreGFP *Wt1* conditionals and ‘phosphoprotein phosphatase activity’ for the *Pax8*^+/Cre^-driven mutants (9 genes, *P*=5.33E–04). For the GO term ‘Cellular Component’, the ‘extracellular region part’ was the most significant for the *Nes*-Cre *Wt1*^co/co^ (42 genes, *P*=2.24E–08), ‘cytosolic ribosomal subunit’ for *Wnt4*^+/CreGFP^ *Wt1*^co/co^ (5 genes, *P*=4.11E–05) and ‘basal plasma membrane’ for the *Pax8*^+/Cre^ *Wt1*^co/c^^o^ mutants (5 genes, 1.11E–04). Correspondingly, the most enriched gene families in the increased genes were ‘Claudins' for the *Nes*-Cre-driven mutants (3 genes, *P*=3.18E–04), ‘ribosomal proteins' for the *Wnt4*^+/CreGFP^ (5 genes, *P*=3.42E–04) and again ‘Claudins' for the *Pax8*^+/Cre^ *Wt1*^co/co^ mutants (3 genes, *P*=5.33E–04). For the decreased genes ‘Molecular Function’ GO terms were most enriched for ‘symporter activity’ for the *Nes*-Cre *Wt1*^co/co^ (9 genes, *P*=9.41E–07), ‘cofactor binding’ for *Wnt4*^+/CreGFP^ *Wt1*^co/co^ (37 genes, *P*=1.84E–17) and ‘transporter activity’ for *Pax8*^+/Cre^ *Wt1*^co/co^ mutants (37 genes, *P*=3.23E-09). Most significant ‘Cellular Component’ GO terms for the decreased genes were ‘apical part of cell’ for the *Nes*-Cre-driven mutants (13 genes, *P*=6.51E–06) and ‘brush border’ for both the *Wnt4*^+/CreGFP^ (20 genes, *P*=1.96E–16)- and *Pax8*^+/Cre^ (11 genes, *P*=2.39E–10)-driven mutants.

To describe the biological consequences of the different developmental blocks in more detail, we analyzed the ‘Biological Process' GO terms and ‘Human and Mouse Phenotypes' terms using ToppCluster. Increased genes (Fig. 3D) function in positive regulation of cell proliferation, circulatory system development and renal system development, and are involved in increased metanephric mesenchyme apoptosis and absent kidneys for all three mutants. Increased genes shared by *Nes*-Cre- and *Pax8*^+/Cre^-driven mutants were involved in the negative regulation of cell death, response to hypoxia and positive regulation of cell migration. Increased genes in the *Nes*-Cre *Wt1*^co/co^ kidneys were uniquely involved in nephron development, kidney size and positive regulation of epithelial-to-mesenchymal transition (EMT), whereas phenotypes uniquely linked to the *Pax8*^+/Cre^ *Wt1*^co/co^ mutants include glomerular crescent and abnormal glomerular capillary morphology. An apparent upregulation of the EMT in the *Nes-*Cre *Wt1*^co/co^ is in accordance with the block in the opposite MET we found in the histological examination of this mutant (Fig. 1B). Decreased genes (Fig. 3E) in all three mutants showed a strong correlation with normal kidney function and physiology, and glomerular development. More specific functions and phenotypes were found to be shared between two mutants or uniquely for single mutants, such as ‘regeneration’ for the *Nes*-Cre-driven mutants, epithelial cell differentiation for the *Pax8*^+/Cre^ *Wt1*^co/co^ mutants (in accordance with this being the only mutant capable of undergoing the normal renal MET) and renal salt wasting shared between the *Nes*-Cre and *Wnt4*^+/CreGFP^ mutants.

### Confirmation of the differential renal phenotypes

**Fig. 4.**
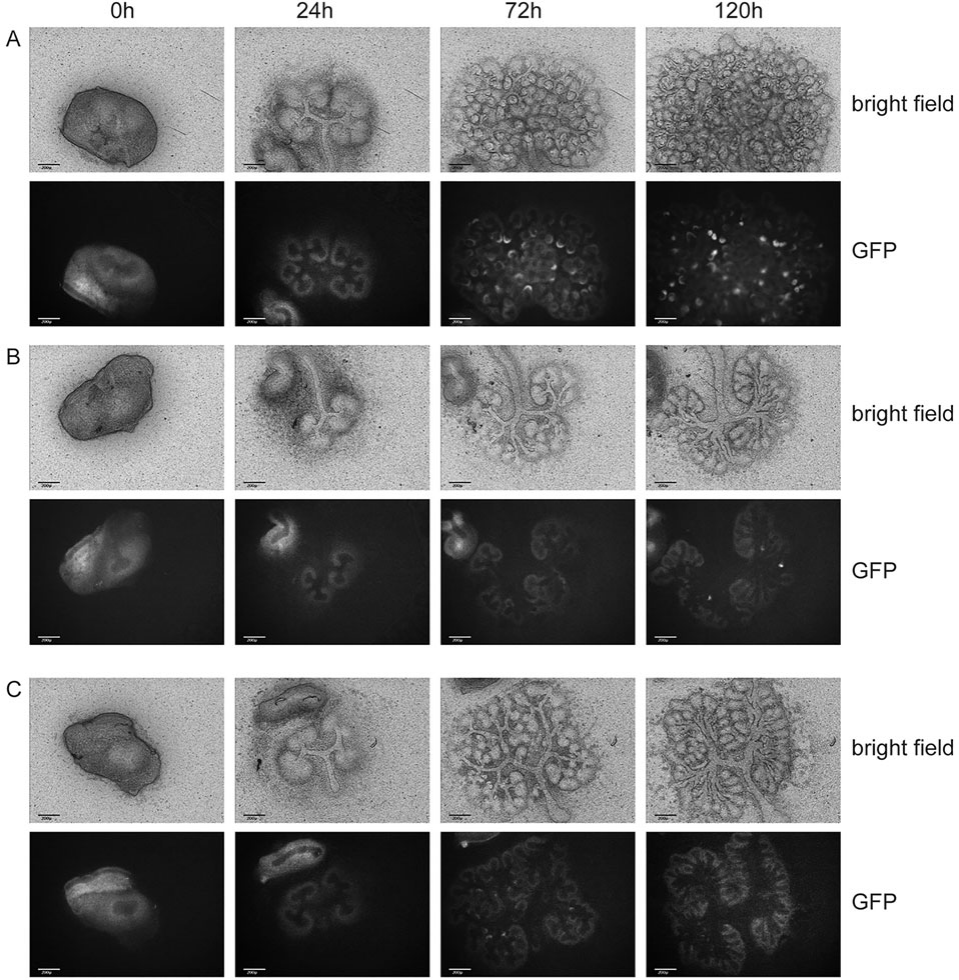
**Time-lapse analysis of control and conditional *Wt1* mutants at the indicated time points.** (A) *Wt1*^co/GFP^ (control). (B) *Nes*-Cre *Wt1*^co/GFP^. (C) *Pax8*^+/Cre^ *Wt1*^co/GFP^. Scale bars: 200 µm.

The combined data showed that *Nes*-Cre-driven loss of *Wt1* results in disturbance of the MET, whereas *Pax8*-Cre *Wt1*^co/co^ reaches a developmental block after the MET but before tubule maturation and glomerulogenesis. *Wnt4*^+/C^^reGFP^ *Wt1*^co/co^ seems to be a combination of both; the reduced epithelialization suggests that, in some cases, the MET is still disturbed, whereas, in cases where the MET was successful, the resulting nephrons were blocked at the same stage as observed in the *Pax8*^+/Cre^ *Wt1*^co/co^ mutants. We therefore limited a more detailed phenotypic analysis to the *Nes*-Cre *Wt1*^co/co^ and *Pax8*^+/Cre^ phenotypes. We first analyzed the nephrogenic progress in control and mutant kidneys on time-lapse using a *Wt1*^GFP^ knock-in allele (Hosen et al., 2007). This reporter allele lacks *Wt1* exon 1, just as the conventional knockout and our conditional allele (after Cre activity) do. We crossed this allele to the *Wt1*^co^ model; in this combination the reporter allele will remain active in the absence of the wild-type allele and can therefore be used to identify, follow and isolate the mutant cells. In control *Wt1*^co/GFP^ kidneys the brightfield data showed clearly how condensed mesenchymes are formed around the tips of the growing ureteric bud, which subsequently epithelialize and form mature nephrons. The GFP signal (identifying *Wt1* expression) was weak but detectable in the metanephric mesenchyme, increased in the cap-mesenchyme and further increased as the tubular stages are reached, with strong signal only remaining in the podocytes of the mature glomeruli (Fig. 4A and supplementary material Movie 2), thereby closely mimicking the known Wt1 expression pattern. Mutant *Nes*-Cre *Wt1*^co/GFP^ kidneys showed the increase in GFP signal in the cap-mesenchyme but no further development (except in some escaping nephrons as discussed before), whereas the brightfield data showed a corresponding lack of epithelialized structures (Fig. 4B and supplementary material Movie 2). The brightfield signal for *Pax8*^+/Cre^ *Wt1*^co/co^ kidneys showed the formation of condensed mesenchymes and some epithelialization and nephron formation, accompanied by an increase in GFP signal in the cap-mesenchyme and a slight further increase as the early nephrons form; however, complete maturation of the signal as seen in the controls was missing (Fig. 4C and supplementary material Movie 2). The phenotypes found in this time-lapse analysis therefore confirm the histological and microarray analysis of the mutants.

**Fig. 5.**
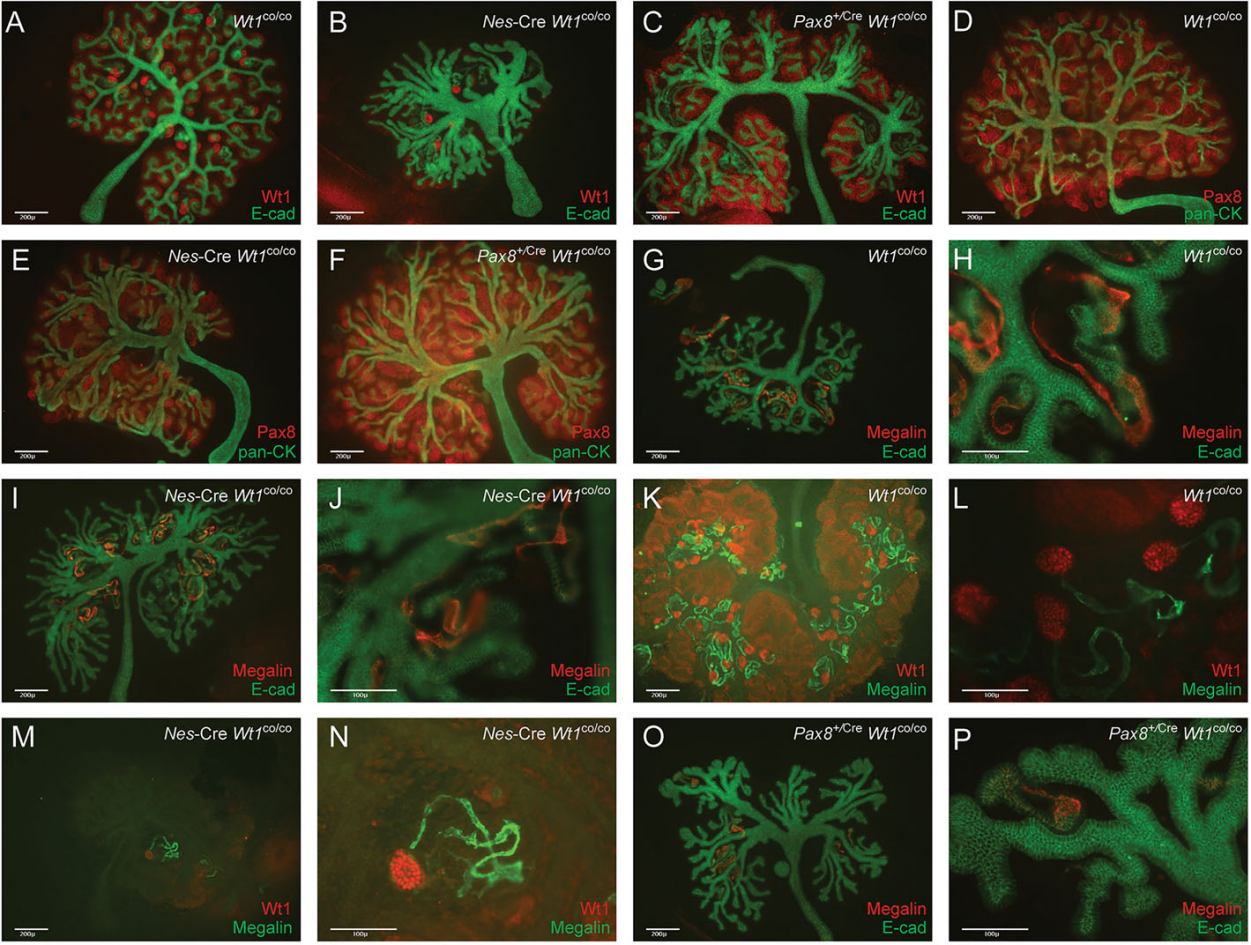
**Antibody staining of cultured kidney rudiments (E11.5+6 days in culture) for Wt1, E-cadherin (E-cad), Pax8, pan-Cytokeratin (pan-CK) and Megalin.** Genotypes and antibodies are indicated.

The phenotypes observed in the E18.5 H&E-stained sections and in the time-lapse analysis were analyzed using antibody staining of kidney organ cultures (Fig. 5). Antibody staining for E-cadherin and Wt1 of control kidneys (*Wt1*^co/co^) showed the expected E-cadherin staining in ureteric bud and developing tubules, the latter confirming the normal MET; Wt1 staining was found in the cap mesenchyme as well as at different stages of tubulogenesis and glomerulogenesis (Fig. 5A). *Nes*-Cre-mediated loss of *Wt1* resulted in an almost complete loss of WT1 staining in the mesenchyme, and the almost complete absence of E-cadherin-positive nephrons confirmed the disturbed MET (Fig. 5B). A few escaping nephrons with increased Wt1 staining indicative of mature podocytes and E-cadherin in the corresponding tubules could easily be identified. In contrast, in *Pax8*^+/Cre^ *Wt1*^co/co^ kidneys, there was a clear increase in Wt1 expression in the cap-mesenchyme as expected for the later loss of Wt1 in this mutant. E-cadherin staining confirmed early stages of tubulogenesis taking place, but Wt1 was lost by the time this stage was reached (Fig. 5C). Using antibodies against Pax8 as a marker for nephron induction and pan-Cytokeratin as an epithelial marker, the same phenotypes were found (Fig. 5D-F). We looked in more detail at the development of the proximal tubules using antibodies against Megalin. Staining for this marker was found in control and *Nes*-Cre *Wt1*^co/co^ samples (Fig. 5G-J) and, as expected, co-staining with anti-Wt1 and -Megalin antibodies confirmed that, in the mutants, Megalin was only found in the escaping nephrons (Fig. 5K-N). In controls, Megalin was found in proximal nephron segments where E-cadherin expression was low. *Pax8*^+/Cre^ *Wt1*^co/co^ kidneys showed expression of Megalin and E-cadherin intermingling, suggesting a patterning defect (Fig. 5O,P).

**Fig. 6.**
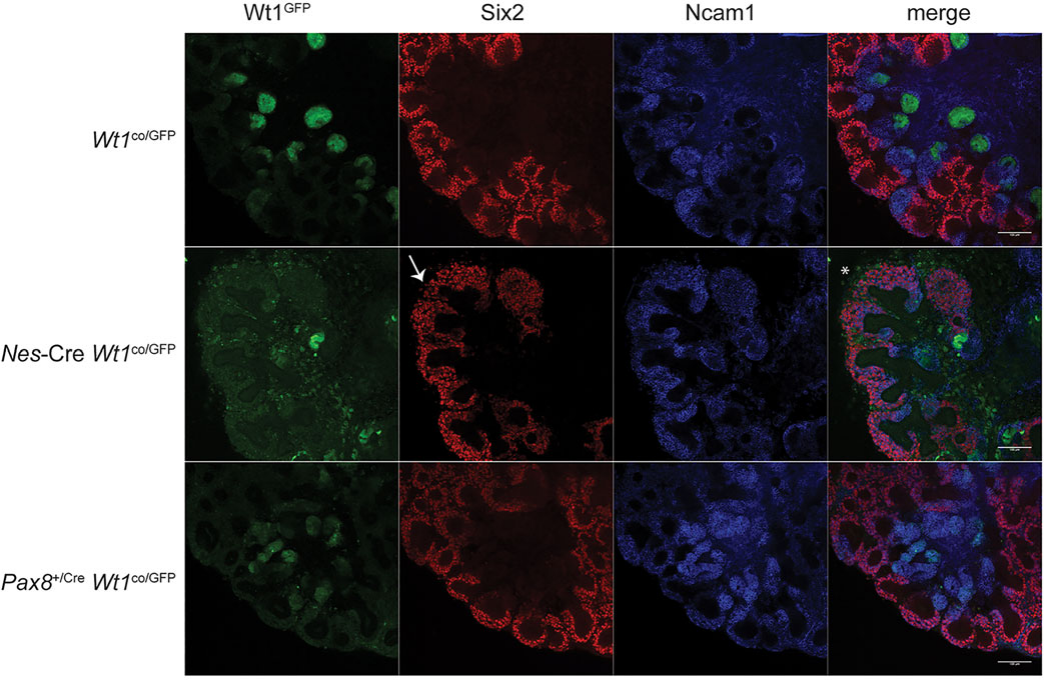
**Antibody staining for nephron progenitor markers (E11.5+6 days in culture).** *Wt1*^GFP^ signal, antibodies and genotypes as indicated. Arrow indicates loose disorganized cap mesenchyme; asterisk indicates mesenchymal *Wt1*^GFP^-positive cells outside the cap mesenchyme. Scale bars: 100 µm.

Wilms' tumour development is in many cases directly linked to a loss of control of the nephrogenic progenitor cells (Hohenstein et al., 2015). In murine embryonic kidneys, Six2 is considered a marker for these progenitor cells (Kobayashi et al., 2008), whereas, in human embryonic kidneys, NCAM1 was identified as a nephron progenitor marker (Harari-Steinberg et al., 2013). We therefore stained the *Nes*-Cre- and *Pax8*^+/Cre^-driven mutants for these markers (Fig. 6). To identify the *Wt1*-mutant cells, we included the *Wt1*^GFP^ allele in this. As shown before (Harari-Steinberg et al., 2013), the expression of Ncam1 overlaps with Six2 expression, but is also found in the epithelialized nephron, especially towards the proximal end. In *Nes*-Cre *Wt1*^co/GFP^ kidneys there was a decrease in the number of Six2^+^ cells, but this is likely linked to the smaller size of these kidneys. However, the Six2^+^ compartment was more disorganized and in some places the signal extended beyond the normal cap mesenchyme that sits closely around the ureteric tip. In addition, there were ectopically located *Wt1*^GFP^-positive cells in the centre of the kidney, most likely corresponding to the extended mesenchyme we identified in the histological analysis (Fig. 2B). These cells are negative for Six2 and Ncam1, suggesting that they are at a stage preceding the progenitor stage or have differentiated to a completely different fate. We also noted that, whereas in the controls there were no *Wt1*^GFP^-positive cells outside the Six2^+^/Ncam1^+^ cap mesenchyme, in the *Nes*-Cre *Wt1*^co/GFP^ kidneys such cells were widespread. Finally, the GFP signal in the cap mesenchyme and the region around it was higher in the *Nes*-Cre-driven mutants than in the controls.

In comparison, the *Pax8*^+/Cre^ *Wt1*^co/GFP^ mutants did not show the GFP signal in the centre of the kidney, consistent with the absence of the extended mesenchyme in these mutants (Fig. 2D), nor did they show the signal outside the cap mesenchyme. Consistent with the histological data, there were Six2^−^/Ncam1^+^ signals that showed an increase in the GFP signal, confirming that these mutants can reach the post-MET/early tubulogenesis stage but cannot form glomeruli. The Six2^+^ cap mesenchyme was normal, in line with the later developmental block compared to the *Nes*-Cre *Wt1*^co/GFP^ mutants.

**Fig. 7.**
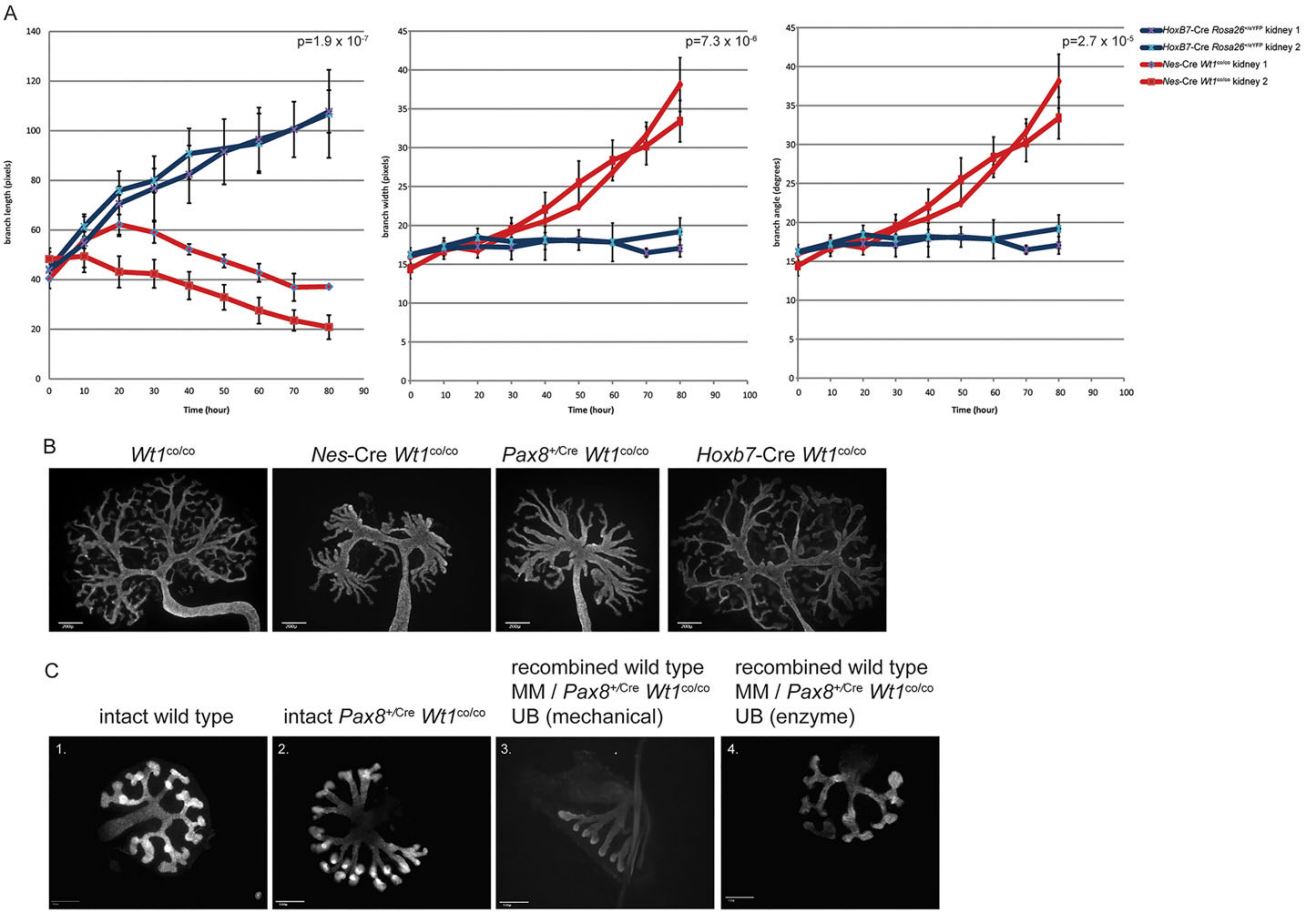
**Branching phenotype in *Wt1* mutants.** (A) Quantification of branch length, width and angle using time-lapse analysis. Two independent mutant and control kidneys were analyzed and shown individually. T=0 is the moment a branch formed. Error bars indicate the s.e.m. of different branches in the same kidney, *n*≥6. *P*-values were calculated using a two-tailed Student's *t*-distribution. (B) Pan-Cytokeratin antibody staining in indicated genotypes (E11.5+6 day culture). Scale bars: 200 µm. (C) Recombination experiments between wild-type mesenchymes and *Pax8*^+/Cre^ mutant ureteric buds stained for calbindin-D-28k antibodies. Scale bars: 100 µm. Panel 1: wild-type kidney (E11.5+2 day culture). Panel 2: *Pax8*^+/Cre^ *Wt1*^co/co^ kidney (E11.5+2 day culture). Panel 3: recombined wild-type mesenchymes with mechanically dissected *Pax8*^+/Cre^ *Wt1*^co/co^ ureteric buds (E11.5+2 day culture). Panel 4: recombined wild-type mesenchymes with mechanically dissected *Pax8*^+/Cre^ *Wt1*^co/co^ ureteric buds (E11.5+2 day culture).

We noticed that, in organ cultures from both mutants, the ureteric buds showed an aberrant branching pattern. Whereas control kidneys showed the expected bifurcation and occasional trifurcation (Watanabe and Costantini, 2004), the mutant ureteric buds showed many more branches apparently coming from the same node. To describe this phenotype in more detail we analyzed the dynamics of the ureteric bud branching using time-lapse imaging. For *Nes*-Cre *Wt1*^co/co^ kidneys, we could use the brightfield signal but, in control kidneys, the development of nephrons rapidly obscured the ureteric bud, so for this we used *Hoxb7*-Cre (Yu et al., 2002) to activate the eYFP reporter specifically in the ureteric bud and analyzed the fluorescent signal. These data showed that the phenotype does not result from one tip giving rise to more than two branches. Instead, the branching develops relatively normally for the first 3 days, after which branches stop elongating and bifurcating further but contract while the nodes move into each other. This was confirmed by quantifying branch length, width and angles in mutant and control kidneys (Fig. 7A; supplementary material Fig. S3).

Because no *Wt1* expression has been reported in the ureteric bud, the observation of a branching phenotype in these mutants was unexpected. Although the *Pax8*^+/Cre^ driver is active in the ureteric bud, the *Nes*-Cre seems not to be active there (Fig. 1), making low-level (but functional) *Wt1* expression in the ureteric bud an unlikely cause of the phenotype. To fully exclude this possibility we crossed the *Wt1* conditional knockout with the *Hoxb7*-Cre driver to knockout *Wt1* in the ureteric bud only. *Hoxb7*-Cre *Wt1*^co/co^ mice were born in the expected ratio, and were viable and healthy (data not shown). *Hoxb7*-Cre *Wt1*^co/co^ showed a normal branching pattern in kidney organ culture (Fig. 7B). This shows that the disturbed branching of the ureteric bud is caused by loss of *Wt1* in the mesenchymal compartment. We sought to rescue the branching phenotype using recombination experiments between *Wt1* mutant ureteric buds with wild-type mesenchymes (Fig. 7C). Mechanical dissection of the mutant ureteric bud followed by recombination with wild-type mesenchymes and subsequent *in vitro* culture still showed the branching defect. However, this mechanical dissection leaves a thin layer of mesenchymal cells (in this case *Wt1*-mutant) attached to the ureteric bud. When these cells were removed using trypsin, the wild-type mesenchyme rescued the branching phenotype (Fig. 7C). This not only confirms that the branching phenotype is caused by loss of *Wt1* in the mesenchymal compartment, it identifies the mesenchymal cells directly lining the ureteric bud as the cells from which the phenotype originates.

### Differential Wt1 phenotypes correspond to different Wilms’ tumour sub-groups

**Fig. 8.**
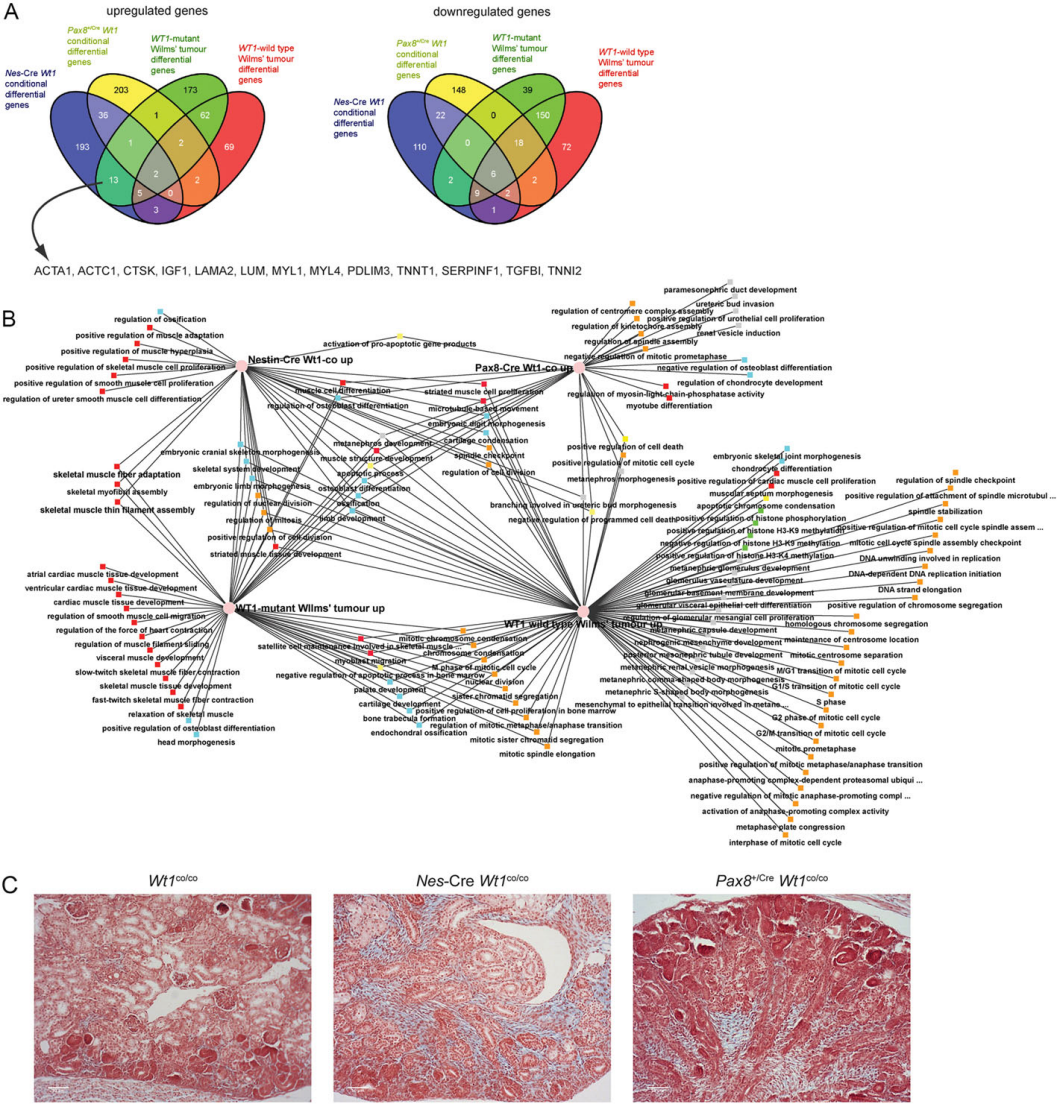
**Comparison of *Nes*-Cre *Wt1*^co/co^, *Pax8*^+/Cre^*Wt1*^co/co^, *WT1*-mutant and *WT1*-wild-type Wilms' tumour microarray data.** (A) Comparison at the gene level. The 13 genes in the *Nes*-Cre *Wt1*^co/co^/*WT1*-mutant Wilms' tumour overlap that give enrichment for muscle functions (see main text) are indicated. (B) Comparison at the GO-term ‘Biological Process'. Red nodes: muscle-related. Light blue nodes: bone/cartilage-related. Yellow nodes: apoptosis-related. Grey nodes: kidney-development-related. Green nodes: histone-modification-related. Orange nodes: cell-cycle-related. (C) Alcian Blue/Alizarin Red staining of E18.5 sections. Scale bars: 50 µm.

Because the *Nes*-Cre and *Pax8*^+/Cre^ drivers result in Wt1 loss immediately before and after the nephron MET, respectively, we reasoned that if disturbance of the MET is indeed important in the formation of Wilms' tumours (Hastie, 1994), characteristics of the tumours might already be found in these E18.5 mutant kidneys. If so, it would suggest that these changes are the direct effects of *WT1* loss rather than events selected for during, or bystander effects of, the tumorigenic process. We compared a published microarray dataset of *WT1*-mutant and *WT1*-wild-type tumours (Corbin et al., 2009) to the *Nes*-Cre *Wt1* conditional and *Pax8*^+/Cre^ *Wt1* conditional datasets (Fig. 8A; supplementary material Table S4). For the decreased genes the biggest overlap was found in the intersection between the *Nes*-Cre *Wt1* conditional and both sets of Wilms' tumour datasets (9 genes) or the *Pax8*^+/Cre^ *Wt1* conditional and both tumour sets (18 genes). We used the ToppGene analysis tool to identify significant functional enrichment in these gene sets. Significant enrichments for both sets were indicative of disturbed kidney function (supplementary material Table S4). For the increased genes, the highest overlap was found between the *Nes*-Cre *Wt1* conditional increased genes and the *WT1*-mutant Wilms' tumour increased genes (13 genes, Fig. 4A). These genes were enriched for muscle-related GO terms (supplementary material Table S4). The ‘Molecular Function’ significant hits included ‘myosin binding’ (*P*=3.15E–6), ‘troponin T binding’ (*P*=2.79E–6) and ‘structural constituent of muscle’ (*P*=3.75E–4). Significant hits for the ‘Biological Process' GO term included only muscle-related terms, like ‘muscle filament sliding’ (*P*=1.00E–13), ‘muscle organ development’ (*P*=9.59E–5) and ‘muscle contraction’ (*P*=2.46E–8). Finally, the ‘Cellular Component’ GO term also identified the ‘muscle’ theme, with significant hits including ‘sarcomere’ (*P*=9.30E–12), ‘striated muscle thin filament’ (*P*=2.46E–7) and ‘troponin complex’ (*P*=1.23E–5).

We analyzed the increased gene sets in more detail at the functional level using ToppCluster for ‘Biological Process' GO terms. Enriched terms were colour coded for different categories to enable the visual recognition of patterns in the datasets (Fig. 8B). In accordance with the comparison at the gene level, muscle-related terms (red nodes) strongly cluster with the *Nes*-Cre *Wt1* conditional and/or *WT1*-mutant Wilms' tumours samples. Within these datasets, a ‘developmental cascade’ can be recognized; terms uniquely enriched in the *Nes*-Cre *Wt1*^co/co^ samples are related to muscle cell proliferation and differentiation, in the overlap between the *Nes*-Cre *Wt1*^co/co^ and *WT1*-mutant tumours for filament assembly and uniquely in the *WT1*-mutant tumours for muscle-function-related terms, such as contraction and relaxation, as well as differentiation into specific muscle types. GO terms related to bone and cartilage processes (blue nodes) do not show such dataset-specific enrichment clustering. Apoptosis-related GO-term enrichment (yellow nodes) are spread over the four datasets as well, but there is a remarkable shift from enrichment of pro-apoptotic GO terms for the two mouse samples to negative regulation of apoptosis in the tumour samples. Enrichment for epigenetic modifier functions (green nodes), especially H3K4 and H3K9 methylation, is uniquely found in the *WT1*-wild-type tumours. Cell cycle/cell division enrichment (orange nodes) is also biased towards the *WT1*-wild-type tumours, although not exclusively. Notably, whereas all samples show enrichment for some cell-division-related GO terms, the *WT1* wild-type tumours show enrichment for regulation of every possible aspect of cell cycle control, including every phase transition, spindle functions, checkpoints, DNA replication and chromosome separation. Finally, GO terms related to kidney development (grey nodes) are found for the *Pax8*^+/Cre^ *Wt1*^co/co^ kidneys and the *WT1*-wild-type Wilms' tumours but not in the *Nes*-Cre *Wt1*^co/co^ and *WT1*-mutant Wilms' tumour samples, consistent with an early, pre-MET, origin of these tumours.

The *Nes*-Cre *Wt1*^co/co^ kidneys showed an expression signature consistent with early muscle differentiation, but an increased differentiation towards bone and cartilage could be recognized in both mutants (Fig. 8B). We therefore analyzed these kidneys with histological stains commonly used to detect bone (Alizarin Red) and cartilage (Alcian Blue). Alizarin Red could be detected throughout both mutant and control kidneys (Fig. 8C); this was much less intense than seen in developing bone in the same sections (not shown). We do not know whether the signal we saw in the kidneys is aspecific or indicates the presence of calcium throughout the control and mutant kidneys. Alcian Blue was not found in the control kidneys, but was seen in the expanded mesenchyme in the *Nes*-Cre *Wt1*^co/co^ throughout the nephrogenic zone and the medulla (Fig. 8C). Some Alcian Blue staining was found in the *Pax8*^+/Cre^ *Wt1*^co/co^ mutant kidneys but only in the medulla and not throughout the nephrogenic zone.

These data demonstrate that the primary results of pre-MET *Wt1* loss, as modelled by the *Nes*-Cre *Wt1*^co/co^ mice, are the earliest event in the development of *WT1*-mutant Wilms' tumours. The *WT1*-wild-type tumours show a closer phenotypic resemblance to the effects of the post-MET *Pax8*^+/Cre^-mediated loss of *Wt1*, even though the initiating genetic event will be different for these tumours. Separating the two types of Wilms' tumours through phenotypic overlap with these two mouse models identifies clear biological differences between the tumour types.

## DISCUSSION

Wilms' tumour is an archetypal example of a tumour that is the direct effect of disturbance of normal development. The developmental origin provides an important platform to describe the earliest events of the tumorigenic process. Although different groups of Wilms' tumours have been described, the genetically best-defined group still remains the 15-20% of cases that are caused by biallelic loss of the *WT1* tumour suppressor gene. To understand how loss of *WT1* causes Wilms' tumours, a better understanding of its role in normal kidney development is essential. To this end, we have used a conditional *Wt1*-knockout mouse model and different Cre drivers, thereby focussing on the MET at the onset of nephrogenesis.

### Wt1 as a ‘master facilitator’ of kidney development

We have previously shown that Wt1 is essential for renal nephron formation (Davies et al., 2004) through direct activation of *Wnt4* (Essafi et al., 2011). Here we provide a detailed study of the *Nes*-Cre *Wt1*^co/co^ phenotype using time-lapse imaging, antibody staining and genome-wide expression analysis. The combination of the lack of epithelialization and extended mesenchyme in E18.5 embryos, the *ex vivo* time-lapse phenotype of the *Nes*-Cre *Wt1*^co/co^, the antibody staining of organ cultures (E-cadherin, Pax8, pan-cytokeratin, Megalin, Six2 and Ncam1), the gene enrichment analysis on the microarray data (all this study) and the loss of direct activation of *Wnt4* (Essafi et al., 2011) leave no doubt that this mutant demonstrates the direct role of Wt1 in the control of the nephron MET. Using the *Pax8*^+/Cre^ driver, we show that loss of Wt1 immediately after the MET leads to a block in nephrogenesis after tubulogenesis is initiated but before tubule maturation and glomerulogenesis take place. Antibody staining for E-cadherin and Megalin showed that, although nephrons start to form in *Pax8*^+/Cre^ *Wt1*^co/co^ mutants, the developing nephrons lose their proximal-distal patterning, with both markers being co-expressed along the axis.

Wt1 is expressed at many stages of kidney development of mouse (Armstrong et al., 1993) and human (Pritchard-Jones et al., 1990) nephrogenesis, and, combined with previously published data, the different models used here show that it might have different, albeit essential, functions at all these stages (Ozdemir and Hohenstein, 2014). In the intermediate mesoderm Wt1 has a pro-survival role, demonstrated using the conventional *Wt1* knockout (Kreidberg et al., 1993). Next, Wt1 controls the MET leading to nephron formation (this study and Essafi et al., 2011). Post-MET, Wt1 is essential for tubule maturation and glomerulogenesis (this study). Wt1 is essential for podocyte function as shown by the development of glomerular sclerosis in mice heterozygous for the conventional knockout (Guo et al., 2002; Menke et al., 2003) and *Wt1*^+/R394W^ (Gao et al., 2004; Ratelade et al., 2010) alleles. Because these mutations are present from early in development, a developmental cause of these phenotypes cannot be excluded. However, the renal phenotype in our adult body-wide conditional *Wt1*-knockout model confirms that *Wt1* is essential for podocyte function and maintenance (Chau et al., 2011).

Given the absence of Wt1 expression in the ureteric bud, the branching phenotype was unexpected. However, the combination of the lack of phenotype in the *Hoxb7*-Cre *Wt1*^co/co^ mutants and the recombination experiments undoubtedly place the origin of this phenotype in the mesenchymal-derived component of the developing kidney. Our time-lapse analysis indicated that the phenotype is the result of dynamic remodelling of an apparent normal branched ureteric bud. We recently presented a ‘node retraction’ mechanism similar to this but at a slightly later time point through which early Y-shaped branches convert to parallel V-shaped branches (Lindstrom et al., 2015). The data presented here could implicate Wt1 in the cells lining the ureteric bud in controlling this morphological change.

We previously showed how Wt1 can control the chromatin state of a complete target locus through the ‘chromatin flip-flop’ mechanism, and suggested it does this to control the accessibility of its target loci to other signals and pathways (Essafi et al., 2011). Based on the accumulating functions of Wt1 in different stages of kidney development as shown here, we extend this to propose a ‘master facilitator’ role for Wt1. Using the chromatin flip-flop, Wt1 could oversee correct development at the chromatin level by allowing some genes to respond to specific signals but preventing other genes to respond to these signals at a given developmental stage. At a later stage though, Wt1 could allow these genes to respond to these signals, if it has altered the chromatin state to a submissive state via the flip-flop mechanism. This model would explain why loss of Wt1 would lead to a block in nephron development at different stages. As shown for the control of *Wnt4* expression, loss of Wt1 locks the target locus at the chromatin level and expression cannot be induced, even if a gene-specific activation signal, like a canonical Wnt signal (Karner et al., 2011; Park et al., 2012), is still present. Vice versa, this model would predict that Wt1 activity is not instructive; even if it opened up a locus, the expression of the locus would still depend on another signal. This way, Wt1 facilitates correct development, making the gene necessary but not sufficient.

The differentially expressed genes in our mutant models will be a mixture of direct and indirect Wt1 targets, as well as changes that reflect the stage at which nephron development was blocked. We focused our analysis on the latter and showed that comparing genome-wide differential gene sets and cell-type-specific expression signatures from the GUDMAP projects is an efficient way of identifying and describing the developmental blocks in the different mutants. Identifying the direct Wt1 targets in the differential gene sets and describing the exact role of Wt1 is far from straightforward and falls outside the scope of the current work. Firstly, Wt1 controls gene expression of target genes in a dichotomous manner: the same target gene can be activated in one tissue and repressed in another, or even in different developmental stages of the same tissue (Essafi et al., 2011). The repetitive nature of nephron induction and the expression of Wt1 in different stages of nephron development means that E18.5 samples, as used here, will consist of cell types in which WT1 can activate and repress the same targets, and will contain cell types in which Wt1 is already lost and those where it is still present. For this reason, comparing differential genes from our analysis to E18.5 Wt1 ChIP-seq data as recently published (Motamedi et al., 2014) does not provide conclusive data on targets of Wt1 in specific cell types that could explain the phenotypes described here, nor could the mode of action of Wt1 (activating or repressing) in these phenotypes be deduced from it. We compared our differential genes to the top 1000 peaks from Motamedi et al. (2014) and identified several strong candidates for direct Wt1 targets in our data (supplementary material Table S2), although conservative use of our array data as well as the ChIP-seq data means that this list is far from complete. Confirmation and correct interpretation of these candidates would require means of specifically isolating the mutant cells from the complex samples described above and is currently not possible.

### Wt1 loss and the origins of Wilms’ tumours

A number of microarray studies have described that Wilms' tumours resemble cells from the developing kidney around the MET stage, and found a clear distinction between the *WT1*/β-catenin mutant and wild-type subsets of tumours (Corbin et al., 2009; Dekel et al., 2006; Fukuzawa et al., 2009; Gadd et al., 2012; Li et al., 2002, 2004). Some of these studies have proposed a different developmental origin for these two subgroups based on expression analysis of established tumours (Fukuzawa et al., 2009; Gadd et al., 2012). Gadd et al. analyzed 224 tumours and proposed that *WT1*/β-catenin mutant tumours originate from the intermediate mesoderm, whereas the *WT1*/β-catenin wild-type tumours would originate from the metanephric mesenchyme. Experimental confirmation of this has so far been lacking. The only existing mouse model for *WT1*-deficient Wilms' tumours is based on a combination of conditional *Wt1* loss with activation of *Igf2* (through loss of H19; Hu et al., 2011). Although tumours with this combination of genetic aberrations can be found in patients, phenotypically they are closer to the *WT1*-wild-type subset than the *WT1*/β-catenin mutant tumours (Gadd et al., 2012). Moreover, this mouse model is driven by a low-dose tamoxifen-controlled ubiquitous activation of Cre, making it difficult to determine the exact developmental stage these tumours arose from.

Given the different development stages where nephron development in our mutant models is blocked, we decided to compare the genome-wide expression patterns of the mice to the two main groups of human Wilms' tumour (*WT1*-mutant and *WT1-*wild-type). This comparison showed a close resemblance between the *Nes*-Cre *Wt1*^co^ mutants and the *WT1*-mutant tumours, especially with respect to the ectopic muscle development signature, whereas the phenotype in the *Pax8*^+/Cre^ *Wt1*^co^ mutant kidneys more resembled the one found in *WT1*-wild-type Wilms' tumours. This would suggest that the latter originates in a developmental block at the same stage as the *Pax8*^+/Cre^-driven mutants even if the causative mutation is different and currently unknown. Whereas our data provides experimental support for the model put forward by Fukuzawa et al. (2009) and Gadd et al. (2012) on different developmental origins of these two groups of tumours, our data suggests different developmental stages than deduced by Gadd et al. from the expression profile of the tumours. The authors suggested an intermediate mesoderm origin for the *WT1*-mutant tumours and a metanephric mesenchyme origin for the *WT1*-wild-type cases. If the *Nes*-Cre *Wt1*^co^ phenotype models the first events in the development of *WT1*-mutant tumours as we propose based on the block before the MET stage, the expanded mesenchyme and the muscle differentiation signature, the stage of origin of *WT1*-mutant tumours must be later than the intermediate mesoderm. It is clear from the conventional knockout that loss of *Wt1* at that stage results in an apoptotic response rather than developmental block (Kreidberg et al., 1993). We serendipitously found a single surviving *Nes*-Cre *Wt1*^co/co^ mouse that developed a stromal predominant Wilms' tumour at 5 months of age in the right kidney. Because this mouse had a larger than usual number of escaping nephrons in the left kidney, we assume that this kidney kept the mouse alive to allow the development of the tumour from the right kidney. We do not present this single case as a mouse model for Wilms' tumours, but it does confirm that an MET block can give rise to these tumours. Equally, the post-MET/early tubulogenesis block found in the *Pax8*^+/Cre^ *Wt1*^co^ kidneys more resembles the epithelial nature of *WT1*-wild-type tumours. So, although order of developmental blocks in our data is the same as proposed by Gadd et al., the exact stages have shifted. Interestingly, a recent analysis of Wilms' tumour cancer stem cells (CSCs) has shown that these cells dedifferentiate to an early developmental stage to form the bulk of the tumour (Shukrun et al., 2014), indicating that the histology (and by extension the expression pattern) of a Wilms' tumour does not necessarily represent the stage of origin of the tumour.

Our analysis of Six2 and Ncam1 expression showed that the expanded mesenchyme we observe in the histological analysis of the *Nes*-Cre-driven *Wt1* mutants is negative for both these nephron progenitor cell markers. This can be explained by either this mesenchyme originating from the nephron progenitor cells but differentiating to a completely different cell fate, or the expanded mesenchyme originating from a stage preceding the nephron progenitor stage. At present we cannot exclude the former, but the latter would be in full accordance with the *Wt1*-mutant tumours originating from an earlier developmental stage. This expanded mesenchyme also stains positive for Alcian Blue and these mutants show an early muscle-development gene signature. Although E18.5 *Nes*-Cre *Wt1*^co^ kidneys do not show histologically recognizable muscle tissue, the recognition of an early muscle-differentiation signature just a week after loss of *Wt1* could suggest that this is a direct effect of this loss that does not require the activating mutations in *CTNNB1* that are found in the majority of these tumours. It could suggest an inhibiting role for Wt1 on muscle development. The literature on this is contradictory. One study described that overexpression of Wt1 in myoblast cells did indeed inhibit their differentiation (Miyagawa et al., 1998), but a second study could not confirm this (Tiffin et al., 2003). The data presented here clearly justifies more work on this. We propose that, prior to the condensation of the mesenchyme to form the cap, the kidney cells still have the potential to form other mesodermal tissues, and Wt1 is stopping them from doing so using the same chromatin flip-flop and master-facilitator mechanisms as discussed above. At later stages the cells become committed to a renal fate, and subsequently Wilms' tumours arising after this step (the *WT1*-wild-type tumours) cannot form the ectopic tissues found in the *WT1*-mutant cases. Of note, although generally the occurrence of ectopic differentiation is considered a characteristic of the *WT1*-mutant subset of tumours, there are cases known which have mutations in *CTNNB1* but not in *WT1*. Instead they lack expression of *WT1* and show a gene expression signature comparable to the *WT1*-mutant tumours (for instance, tumour set WT-A2 in Corbin et al., 2009). One could argue that these tumours are in effect the same as the *WT1*-mutant tumours, having no functional WT1 protein in combination with a *CTNNB1* mutation. Although the initiating event would be different (and currently unknown) from the *WT1*-mutant subset, the developmental stage at which this initiating event would occur would be the same as in *WT1*-mutant tumours. For instance, a gene upstream of *WT1* expression at this stage could be affected, leading to this phenotype. Alternatively, the dedifferentiation of the Wilms' tumour CSCs as described by Shukrun et al. (2014) could play a role in the ectopic differentiation found in these tumours. Either way, better understanding of this group of tumours could provide valuable new insights into the link between stage of origin and Wilms' tumour phenotype.

If our interpretation of the *Nes*-Cre- and *Pax8*^+/Cre^-driven *Wt1* mutants and how they compare to *WT1*-mutant and *WT1*-wild-type tumours is correct, our data also highlights other differences between these two tumour groups. Most remarkable is the presence of classic cancer hallmark GO terms in the *WT1*-wild-type tumour/*Pax8*^+/Cre^ *Wt1* conditional sets but absence of these in the *Nes*-Cre-driven mutants/*WT1*-mutant tumours. A picture emerges where the *WT1*-mutant tumours start as a purely developmental problem, almost like a teratoma that is restricted to the mesodermal lineage, whereas the *WT1*-wild-type tumours are more classical cancers right from the start. This difference, if correct, would mean that very different treatment regiments would be needed to treat these different tumour groups. Indeed, with the present therapies, the *WT1*-mutant subset of tumours is much better treatable than their *WT1*-wild-type counterpart.

In conclusion, we have generated a developmental series of renal *Wt1* mutants. The data presented here identify the MET and early tubulogenesis stages as developmental steps under control of *Wt1*, suggesting a role as ‘master facilitator’ of kidney development. Comparison of the mutant mouse kidney expression data to Wilms' tumour data is consistent with a block in MET as the origin of *WT1*-mutant Wilms' tumours and highlights clear biological differences between these tumour types.

## MATERIALS AND METHODS

### Mouse lines

All animal experiments were approved by the University of Edinburgh ethical committee and according to Home Office legislation. Animal models used were the following: Wt1^tm1.1Ndha^ (*Wt1* conditional; Martinez-Estrada et al., 2010); Wt1^tm1^^.1Ndha^. *Wt1*-GFP (Hosen et al., 2007); Wt1^tm1Nhsn^. *Nes*-Cre (Tronche et al., 1999); Tg(Nes-cre)1Kln. *Pax8*^+/Cre^ (Bouchard et al., 2004); Pax8^tm1(cre)Mbu^. *Wnt4*^+/CreGFP^ (Shan et al., 2010); Wnt4^tm2(EGFP/cre)Svo^. Hoxb7-Cre (Yu et al., 2002); Tg(Hoxb7-cre)13Amc. *Rosa26*^+/eYFP^ (Srinivas et al., 2001); Gt(ROSA)26Sor^tm1(EYFP)Cos^. Germline Cre mice were a kind gift from Dr D. J. Kleinjan, IGMM, University of Edinburgh. All experiments were done using mice of mixed C57BL/6/129Ola background with varying generations backcrossing to C57BL/6.

### Immunohistochemistry

Embryos were taken at E18.5 and fixed in freshly prepared 4% paraformaldehyde overnight at 4°C. Following fixation the embryos were paraffin embedded, sectioned at 7 μm and stained with haematoxylin/eosin. Alcian Blue and Alizarin Red staining was done as described elsewhere (Bancroft and Stevens, 1990).

### Kidney organ cultures

Kidneys at the T-bud stage of development were isolated from E11.5 embryos and cultured on 0.4 µm pore size Transwell filters in Minimum Essential Medium Eagle medium with 10% newborn calf serum, 1% penicillin and streptomycin. For antibody staining kidneys were fixed in ice-cold methanol for 10 min, washed briefly in PBS and blocked in PBS, BSA-Azide overnight at 4°C. Primary antibodies [Pax8 (ProteinTech, cat. 10336-1-AP) 1:200, Pan-Cytokeratin (Sigma, cat. C2562) 1:800, E-Cadherin (BD Biosciences, cat. 610182) 1:800, WT1-C19 (Santa Cruz, cat. sc192) 1:200, Megalin (kindly given by Prof. Thomas Willnow, MDC-Berlin) 1:1600, Six2 (LifeSpan Biosciences, LS-C10189) 1:100, Ncam1 (Sigma, cat. C9672) 1:100] were incubated overnight at 4°C. The following day six 1 h washes in PBST (500 ml PBS+500 μl 10% Triton) were carried out at room temperature. Secondary antibodies [Alexa-Fluor donkey anti-rabbit 594, 1:400 (Invitrogen, cat. A21207), Alexa-Fluor donkey anti-mouse 488 1:400 (Invitrogen, cat. A21202) and Alexa-Fluor goat anti-mouse IgG1 (γ1) (for Ncam1 staining, Invitrogen A-21240)] were incubated overnight at 4°C, followed by six 1-h washes in PBST, as above. The kidneys were mounted in Vectashield mounting medium for fluorescence (Vector Labs, cat. H-100). Immunofluorescence was observed and recorded on an imaging system comprising a Coolsnap HQ CCD camera (Photometrics Ltd, Tucson, AZ) and Zeiss Axioplan II fluorescence microscope with Plan-neofluar objectives of a Nikon A1R confocal microscope. Image capture and analysis were performed using in-house scripts written for IPLAB Spectrum (Scanalytics Corp., Fairfax, VA) or Fiji/ImageJ.

The Time Lapse Imaging system comprised a Zeiss Axiovert 200 fluorescence microscope (Carl Zeiss, Welwyn, UK), Lambda LS 300W Xenon source and 10-position excitation and emission filter wheels (Sutter Instruments, Novato, CA) populated with a Chroma #86000 filter set (Chroma Technology Corp., Rockingham, VT), ASI PZ2000 3-axis XYZ stage with integrated piezo Z-drive (Applied Scientific Instrumentation, Eugene, OR), Photometrics Coolsnap HQ_2_ CCD camera (Photometrics, Tucson, AZ) and Solent Scientific incubation chamber with CO_2_ enrichment (Solent Scientific, Segensworth, UK). Image capture was performed using Metamorph software (Molecular Devices, Sunnyvale, CA).

The measurements were taken every 10 h using IPLAB image analysis software (Scanalytics, MD, USA) on the still images obtained from the time-lapse movies. In order to synchronize the measurements between different kidneys and between different branches, the moment that the branch of interest bifurcated was redefined as time 0.

### Microarray analysis

Total RNA was purified from E18.5 kidneys using RNAeasy micro columns and on-column DNase treatment (Qiagen). Samples were labelled using the Illumina^®^ TotalPrep™ RNA Amplification Kit (Life Technologies) and analyzed on Ref8 v2 BeadChips (Illumina). Data was analyzed using Genespring. All samples were analyzed in biological triplicates except for *Nes*-Cre *Wt1*^co/co^ where one sample failed the analysis QC. Overlaps in differentially expressed genes between different mutants were identified using Venny (http://bioinfogp.cnb.csic.es/tools/venny/index.html). Gene-set enrichment analysis was done using ToppGene (http://toppgene.cchmc.org/) and ToppCluster (http://toppcluster.cchmc.org/). Networks were manually ordered, categorized and coloured in Cytoscape 3.02 (Smoot et al., 2011). Microarray genomics data can be found under GEO dataset record GSE70892.

## Supplementary Material

Supplementary Material
