# Supplementary material for: Deducing the stage of origin of Wilms' tumours from a developmental series of Wt1-mutant mice
Source: Dis Model Mech. 2015 Aug 1;8(8):903–17. doi: 10.1242/dmm.018523 (PMC4527280; doi:10.1242/dmm.018523)
Supplement: Supplementary Material [file supp_8_8_903__index.html]

Supplementary Material 

# Deducing the stage of origin of Wilms' tumours from a developmental series of *Wt1*-mutant mice

## DMM018523 Supplementary Material

- Supplementary Material
