# Supplementary material for: Deducing the stage of origin of Wilms' tumours from a developmental series of Wt1-mutant mice
Source: Dis Model Mech. 2015 Aug 1;8(8):903–17. doi: 10.1242/dmm.018523 (PMC4527280; doi:10.1242/dmm.018523)
Supplement: Supplementary Material [file supp_018523_DMM018523supp.pdf]

Supplementary Figures

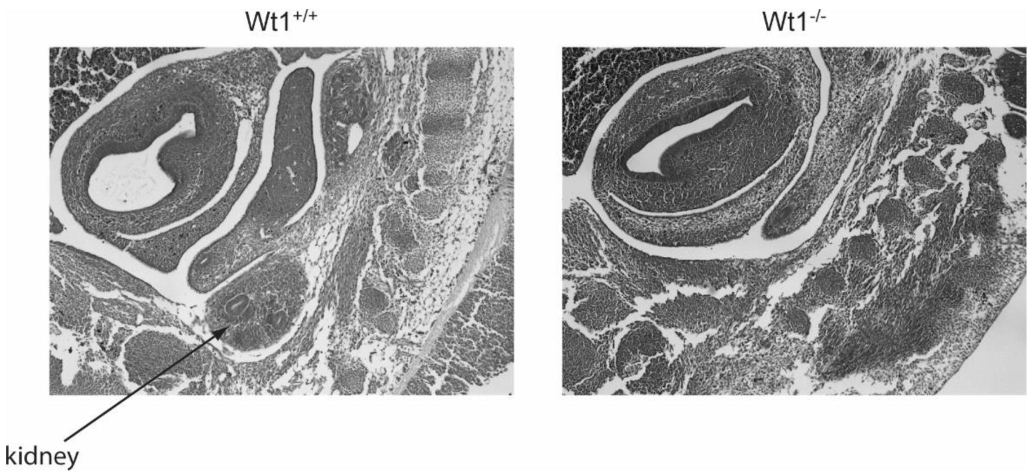

Fig S1. H&E staining of E12.5 embryo sections of wild-type (*Wt1*$^{+/+}$) and homozygous *Wt1* conditional embryos after deletion of *Wt1* using a germline Cre deleter strain (*Wt1*$^{-/-}$).

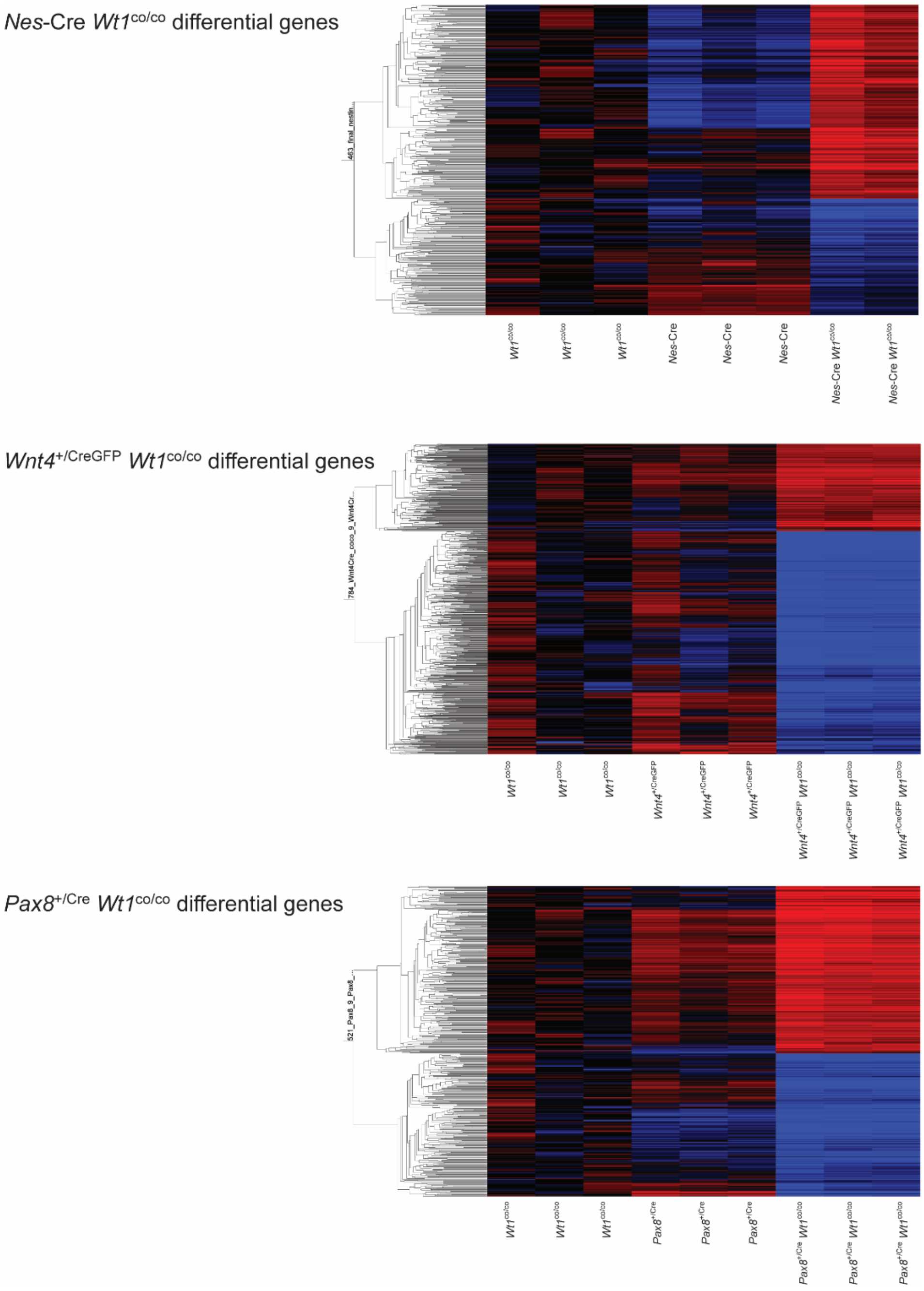

Fig S2. Identification of differentially expressed genes in *Nes*-Cre *Wt1*$^{co/co}$, *Wnt4*$^{+/CreGFP}$ *Wt1*$^{co/co}$ and *Pax8*$^{+/Cre}$ *Wt1*$^{co/co}$ from E18.5 total kidney samples.

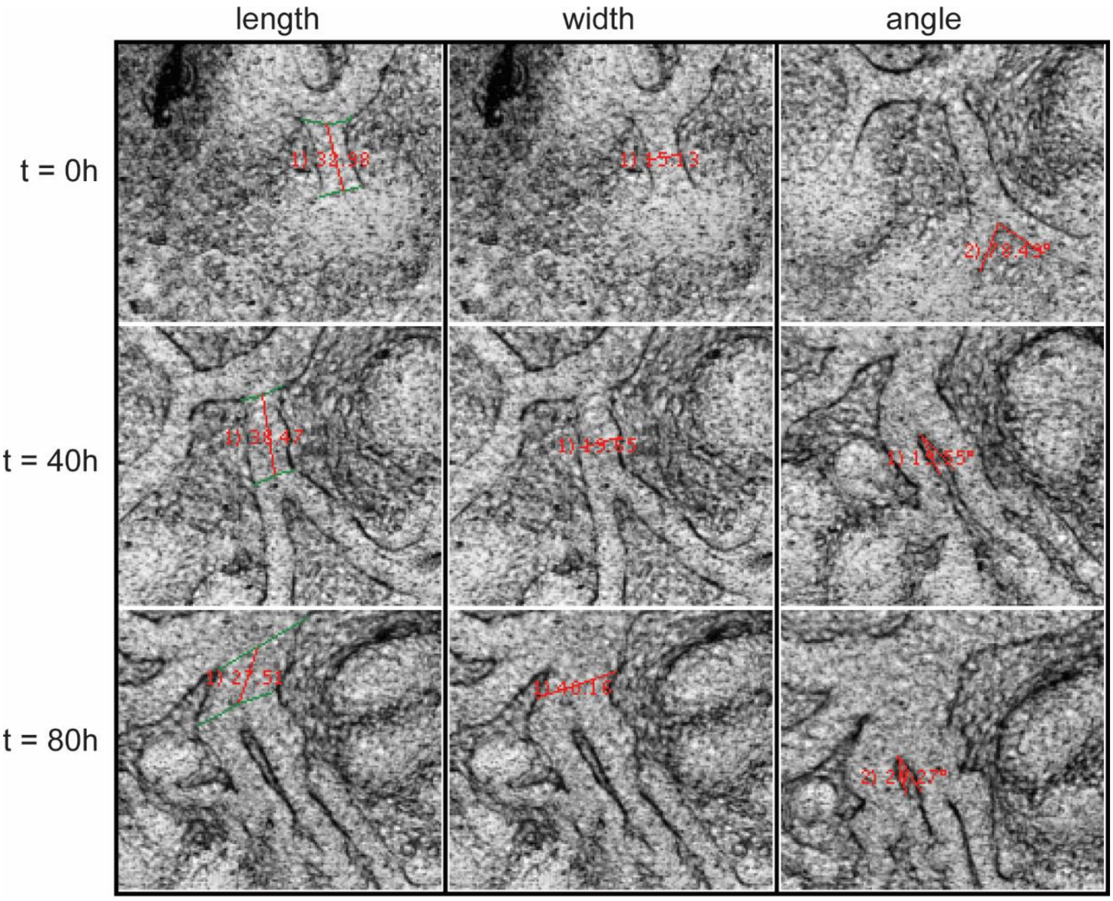

Fig S3. Example of length, width and angle measurement of the same branch at three different time points.

Supplementary movies

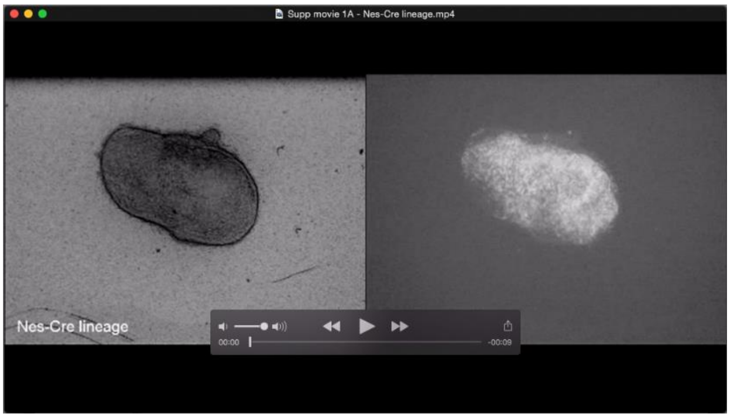

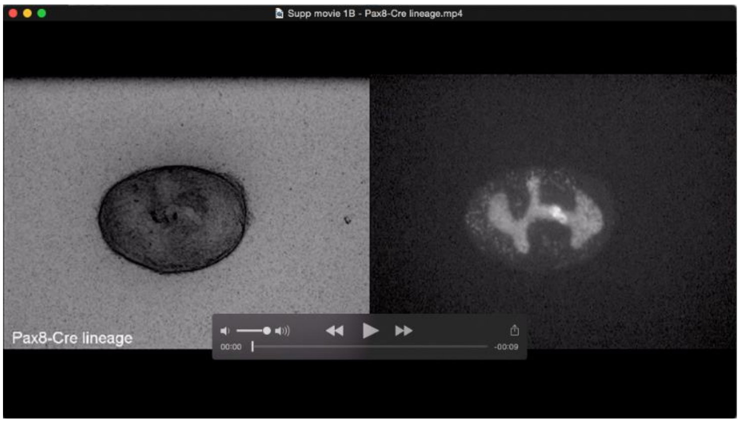

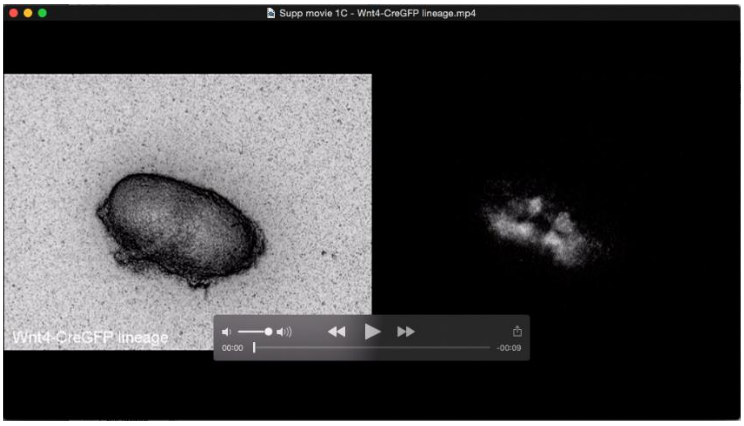

Movie S1. Renal time-lapse lineage tracing of the three Cre drivers crossed using *Rosa26*^{eYFP}. Left: brightfield channel; Right: FITC channel (eYFP signal).

A. *Nes*-Cre lineage tracing
B. *Pax8*$^{+/Cre}$ lineage tracing
C. *Wnt4*$^{+/CreGFP}$ lineage tracing

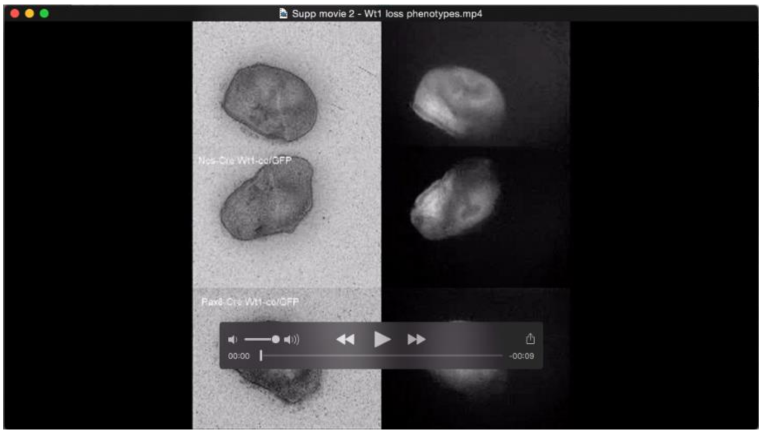

Movie S2. Time-lapse analysis of mutant *Wt1* renal phenotypes.

Supplementary Tables

Table S1. Differentially expressed genes.

Click here to Download Table S1

Table S2. Candidate direct Wt1 targets

Click here to Download Table S2

Table S3. Geneset Enrichment Analysis increased and decreased genes

Click here to Download Table S3

Table S4. Mutant mouse kidney – human tumour comparison

Click here to Download Table S4
